# *“If I use pad, I feel comfortable and safe”*: a mixed-method analysis of knowledge, attitude, and practice of menstrual hygiene management among in-school adolescent girls in a Nigerian city

**DOI:** 10.1186/s12889-024-19256-5

**Published:** 2024-06-27

**Authors:** Nkemdilim Ene, Obasanjo Afolabi Bolarinwa, Chris Adedigba, James Oyeleye, Israel Boboye, Union Nwosu, Fayokemi Olususi, Philip Oluwayemi, Sylvester Reuben Okeke

**Affiliations:** 1 Preston Associates International Development Limited, Plot 1854 Mahathir Mohammed Street, off TY Danjuma Street, Asokoro, Abuja, Federal Capital Territory Nigeria; 2 The Department of Public Health, York St. John University, London, UK; 3 Demography and Population Studies Programme, Schools of Public Health and Social Sciences, University of the Witwatersrand, Johannesburg, South Africa; 4 Centre for Social Research in Health, and School of Population Health, UNSW Sydney, Sydney, Australia; 5 Sydney School of Health Sciences, University of Sydney, Sydney, Australia

**Keywords:** Menstrual hygiene management, Adolescent girls, Knowledge, Attitude, Practice, Mixed-method

## Abstract

**Background:**

Adolescence is a pivotal stage in human development that presents unique challenges, especially for girls navigating the complexities of menstruation. Despite the importance of menstrual hygiene management for adolescent girls’ well-being, this vital aspect of personal health is often overlooked, particularly in regions where cultural stigma prevails. This study examines knowledge, attitude, and practice of menstrual hygiene management among in-school adolescent girls in Abuja, Nigeria.

**Methods:**

The study employed a cross-sectional mixed-method design, integrating quantitative surveys with focus group discussions. A survey was conducted among 420 adolescent girls across four government junior secondary schools through a multistage sampling technique. Also, Focus Group Discussions were conducted among 80 respondents in groups of 10 discussants. The quantitative data set was subjected to descriptive and inferential statistical analysis, while the qualitative data were analysed using content analysis.

**Results:**

Findings revealed that the majority (53.45%) of the respondents had good knowledge of menstruation and menstrual hygiene management. Junior Secondary School (JSS) 3 students [OR = 2,09; 95% CI = 1.24–3.52] and those who started menstruation at age 15 years and above [OR = 7.52; 95% CI = 1.43–39.49] were associated with increased odds of having good knowledge of menstrual hygiene management. The attitude of most respondents (70.08%) towards menstrual hygiene management was good. Those in the JSS 3 class [OR = 6.47; 95% CI = 3.34–12.54], respondents who are Muslim [OR = 2.29; 95% CI = 1.63–5.48], and those whose parents had tertiary education [OR = 3.58; 95% CI = 1.25–10.25] were more likely to demonstrate more positive attitudes compared to their counterparts whose parents do not have tertiary education. In relation to practice, about 3 in 5 (57.80%) reportedly practise good menstrual hygiene management. Respondents who practice traditional religion [OR = 0.33; 95% CI = 0.02–4.56] were less likely to practise good menstrual hygiene management, while respondents who are the third child of their parents [OR = 2.09; 95% CI = 1.04–4.23] were more likely to practise menstrual hygiene compared to respondents with other birth orders. Qualitative results showed that participants had good knowledge of menstruation and menstrual hygiene management, and mothers were the main source of menstruation-related information. Participants had mixed feelings and reactions during their first menstruation, with 3 in 5 participants reporting experiencing menstruation-related stigma restrictions when menstruating.

**Conclusions:**

In-school adolescent girls in Abuja, Nigeria, have good menstruation-related knowledge and positive attitudes, as well as practise menstrual hygiene management. Students’ class and age at first menstruation were major factors associated with good knowledge of menstruation and menstrual hygiene management; respondents’ class, religion and parents’ educational qualification were associated with a positive attitude, while respondents’ religion and parity line were associated with menstrual hygiene practice. Future interventions should focus on conducting school and community-level awareness programs to increase knowledge and dispel myths and misconceptions about menstruation and menstrual hygiene management.

**Supplementary Information:**

The online version contains supplementary material available at 10.1186/s12889-024-19256-5.

## Background

Adolescence is a unique stage of human development that indicates a transition from childhood to adulthood [1]. For girls, adolescence is associated with rapid puberty development and sexual maturation [2]. Female adolescents experience menstruation from puberty throughout their reproductive life, and the beginning of menstruation – menarche – signals a transition to womanhood [3]. Menstruation is the expulsion of blood and tissue from a woman’s reproductive organ due to the lack of conception [4], and it occurs on average every 28 days (a phenomenon termed the menstrual cycle). Hence, menstruation is a normal physiological process that occurs in adolescent girls and women, starting with menarche till menopause [5].

Menstruation is an issue reproductive-age women encounter throughout their reproductive life because it affects their reproductive health, emotions, and productivity to a large extent [6]. About 26% of the global population of females are of reproductive age, and most menstruate for about 3–7 days every month [7]. Also, evidence has shown that an average of 300 million women around the world menstruate daily [7, 8]. The monthly occurrence of menstruation in females of reproductive age amplifies the need for proper menstrual hygiene management. According to the WHO and UNICEF Joint Monitoring Programme (JMP), Menstrual Hygiene Management (MHM) refers to the practices and strategies that women and adolescent girls utilise to effectively manage their menstruation. This includes using clean, absorbent materials to collect menstrual blood, changing these materials regularly, practising proper hygiene by washing the body with soap and water and having access to safe and convenient facilities for proper disposal of used materials [9].

Good menstrual hygiene is essential for all women’s well-being, yet it is still a neglected issue in most parts of the world. In most traditional African cultures, menstruation is considered a taboo and humiliating topic that is rarely discussed publicly [10]. As a result, most adolescent females, particularly in developing countries, are unaware of the need for healthy MHM [11]. Also, MHM is not adequately addressed in the curriculum of most secondary schools, and some girls may lack access to adequate menstrual hygiene facilities. These girls are unable to obtain or purchase sanitary materials to manage their menstrual flow, and so they rely on sub-standard products such as fabric and cotton wool, among others [12]. The United Nations Children’s Fund [13] noted that “lack of menstrual hygiene management negatively impacts girls’ education and health, and girls who manage their periods poorly are more likely to miss school or drop out, leading to negative impacts on their academic and economic potential.”

In recent years, there has been an increasing recognition of the importance of MHM in addressing the needs and rights of women and girls, especially in Low- and Middle-Income Countries (LMICs), to manage their menstrual health safely and comfortably. This is due to global evidence of a lack of adequate guidance, facilities, and materials for girls to manage their menstruation in school [14]. In addition, there are many barriers school girls face regarding safe, hygienic, and dignified menstruation [15]. These barriers contribute to gender discrimination in school environments [16] and pervasive menstruation-related stigma, enabling behavioural restrictions and feelings of shame, stress, and taboo [17].

In Nigeria, factors influencing menstrual hygiene management among secondary school girls include age, educational status, parents’ educational status, family size, residence, and lifestyle [18]. Additionally, monthly family income, lack of hygiene facilities in schools, lack of privacy in school toilets, limited menstrual hygiene education, and the fear of being harassed by boys also play significant roles [19]. Moreover, cultural taboos and stigma surrounding menstruation further contribute to the lack of positive attitudes towards menstrual hygiene management among secondary school girls in Nigeria [20]. For girls to live healthy, productive, and dignified lives, it is essential that they can manage menstrual bleeding effectively. This requires access to clean and adequate water, sanitation, and hygiene services, having somewhere private to change clothes or disposable sanitary pads, and facilities to dispose of used clothes and pads [21]. Failure to provide menstrual hygiene facilities at home or the school level also decreases the level of good menstrual hygiene practices among female students [22].

This paper shares learning outcomes from the baseline assessment of the “2023 Pad A Girl Project” organised by Preston Development Foundation (PDF), Abuja, Nigeria, for adolescent girls in selected Government Junior Secondary Schools (JSS) within the Federal Capital Territory (FCT), Nigeria to commemorate the 2023 World Menstrual Hygiene Day with the theme “Making Menstruation a Normal Fact of Life by 2030”. The project centred on raising awareness about menstrual hygiene management and supplying sanitary products (disposable sanitary pads) to adolescent girls in selected schools.

## Methods

### Study setting

The study was carried out at four selected Junior Secondary Schools (JSS Area 1, JSS Jabi, JSS Kuje, and JSS Pasali) under the umbrella of the Federal Capital Territory- Universal Basic Education Board (FCT-UBEB), Abuja, Nigeria. The choice of the location was informed by the dearth of literature on menstrual hygiene management knowledge and practice among adolescents in the territory. The Federal Capital Territory was created in 1976 from parts of the states of old Kaduna, Kwara, Niger, and Plateau, with the bulk of land mass carved out of Niger state. It is geographically located at the centre of the country with a landmass of approximately 7,315 km^2^, situated within the savannah region with moderate climatic conditions. Unlike other states of Nigeria, which are headed by elected Governors, the FCT is administered by the Federal Capital Territory Administration, headed by a minister whom the president appoints. The FCT-UBE Board was established following the enactment of the UBE Law of 2004 with the mandate to provide quality Basic Education, which includes Early Childcare, Primary, Junior Secondary, and Nomadic Education, to the teeming school-age population in the FCT. Currently, there are 161 Junior Secondary Schools under the board with an enrolment figure of 116,585 students [23].

### Study design

A cross-sectional mixed-methods research design was adopted for this study. This entailed a single-point survey and Focus Group Discussion (FGD) to capture the current state of knowledge, attitude, and practice of MHM among the study sample. The research design was adopted because it allows the generalisation of findings obtained from the sample to the population [24].

#### Sample recruitment and selection

The population for this study comprised 81,439 female students in all 161 government Junior Secondary Schools in the FCT^23^. The sample size was determined using Yamene’s sample size formula with the total population of 81,439 adolescent girls at a confidence level of 95% and a +/-5% margin of error as follows;

1 $$n = \frac{N}{{1 + N * {{(e)}^2}}}$$

n - the sample size

N - the population size

e - the acceptable sampling error

* 95% confidence level and *p* = 0.5 are assumed

In addition, to mitigate against a low response rate, an additional 10% (*n* = 38) of the sample size was added to serve as a buffer. Therefore, a total of 420 respondents were sampled using a multistage sampling technique.

In stage one, all government Junior Secondary Schools were listed, after which the RAND function in Microsoft Excel software was used to select four schools (JSS Area 1, JSS Jabi, JSS Pasali, and JSS Kuje). In stage two, a proportionate sampling technique was used to distribute the total sample (420) across the four selected schools based on the population of each school. Based on this, 40, 114, 173, and 93 respondents were distributed to JSS Area 1, JSS Jabi, JSS Pasali, and JSS Kuje respectively (Table 1). Similarly, in stage 3, a proportionate sampling technique was used to select respondents from each school based on the population of each class. This was achieved by obtaining the list of adolescent girls each from JSS 1–3 and then calculating the proportion based on sample distribution from stage two.

**Table 1:** Sample distribution for quantitative survey

S/*N*	School	Population of Girls	Proportionate sample
1	JSS Area 1	281	40
2	JSS Jabi 1	798	114
3	JSS Kuje	651	93
4	JSS Pasali	1211	173
Total		**2941**	**420**

In addition to the 420 respondents for the quantitative survey, FGDs were conducted for eight (8) groups (two per school) of adolescent girls. Each group consisted of eight (8) to 10 participants randomly selected from other eligible adolescent girls who did not participate in the quantitative survey (a total of 80 adolescent girls).

The main inclusion criteria included being in the post-menarche adolescent girls group, providing consent to participate and getting parental approval to participate in the study.

### Data collection instrument

For the quantitative method, a close-ended questionnaire titled “Questionnaire on Knowledge, Attitude, and Practice of Menstrual Hygiene Management among Junior Secondary School Adolescent Girls developed in the English Language was used. The instrument comprises four sections. Section A: socio-demographic characteristics of respondents; Section B: Knowledge of menstruation and MHM among adolescent girls; Section C: Attitude towards MHM among adolescent girls; and Section D: MHM practices among adolescent girls. In addition, an FGD guide was developed for qualitative data collection. To ensure the validity of the instrument, the quantitative data collection tools were quality assured by three experts to ascertain the appropriateness of questions and/or response wording and sequencing, time allowed, comprehension, and potential recall bias of the tool. Also, to test the reliability of the quantitative instrument, a pilot test was conducted among 20 female students of JSS Kpaduma, while internal consistency reliability was determined using Cronbach’s alpha test, which yielded a 0.76 reliability coefficient.

### Method of data collection

Data for this study was collected using self-administered questionnaires and FGD sessions. The Preston Development Foundation (PDF) staff and trained data collectors administered the questionnaires and conducted the FGDs sessions. The respondents were required to tick the appropriate responses that best represented their views and practices. All administered questionnaires were collected on the spot.

### Data analysis

Upon completion of data collection, quantitative data was collated, coded and analysed using Microsoft Excel and Stata software. Descriptive statistics of frequencies and percentages, mean, and stan was used to analyse the socio-demographic variables of respondents. To assess knowledge, attitude, and practice levels of respondents, a numeric scoring pattern was used, and outcome (dependent) variables – knowledge, attitude, and practice were computed. The outcome variables were further categorised as binary (good or poor) based on mean scores as cut-off marks (Table 2). Respondents who scored greater than the mean scores for knowledge (22.7 ± 3.16), attitude (5.0 ± 0.80), and practice (31.9 ± 3.6) were deemed to be good responses and vice versa.

Furthermore, inferential statistic of chi-square was used to test for association between demographic variables and outcome variables (knowledge, attitude, and practice) at a 95% confidence interval. In addition, the qualitative data was analysed using Atlas.TI (version 22) software. This was performed following the contextual procedure of extraction of audio files from the tape recorder to a laptop for proper labelling, identification, and documentation. Transcription of the interviews was done in English Language before exporting to Atlas.TI software for coding and content analysis.

**Table 2:** Description of scores obtained by respondents (*n* = 391)

Outcome Variables	Maximum Obtainable score	Minimum obtainable score	Mean ± SD	Good *n* (%)	Poor *n* (%)
Knowledge	29	11	22.7 ± 3.16	209 (53.45%)	182 (46.55%)
Attitude	6	3	5.0 ± 0.80	274 (70.08%)	117 (29.92%)
Practice	40	21	31.9 ± 3.6	226 (57.80%)	165 (42.20%)

## Results

### Outcome variables

**Fig. 1:**
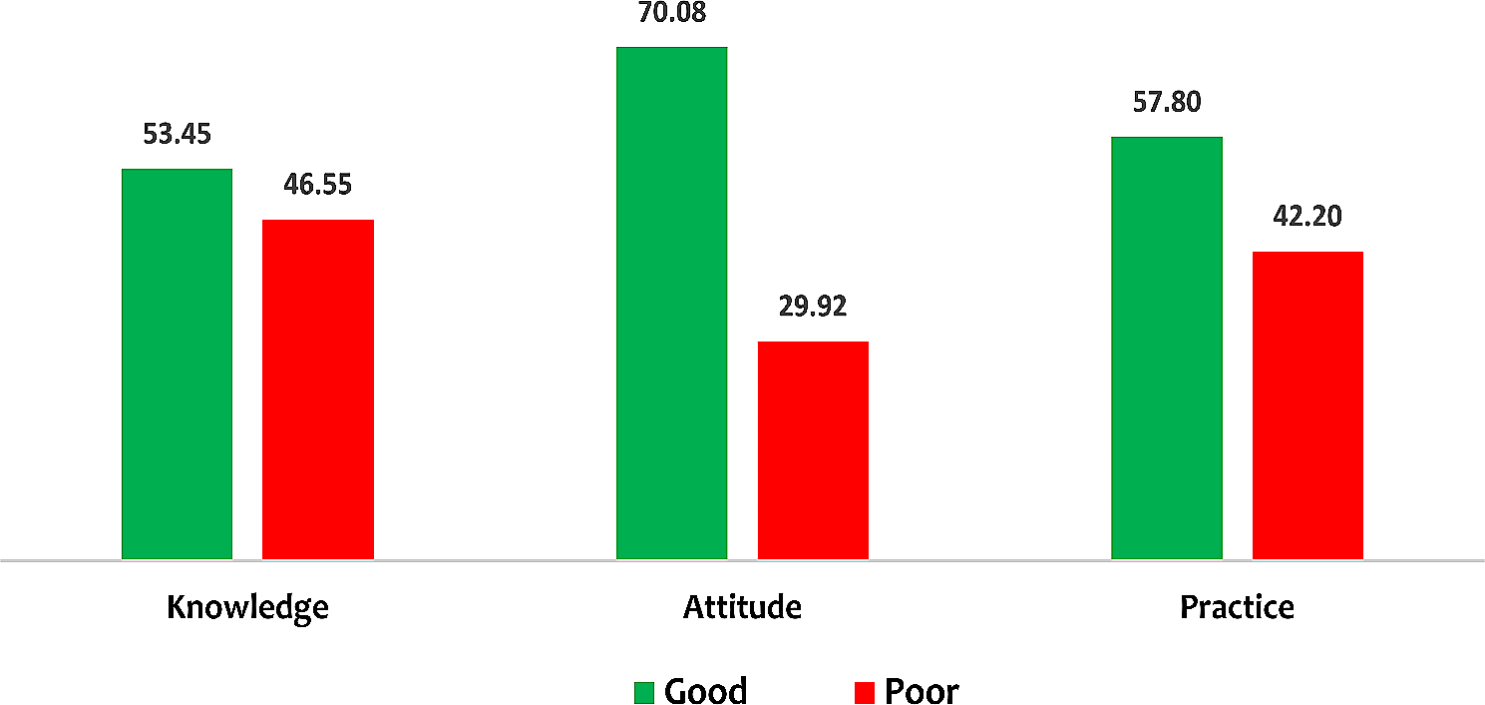
Percentage distribution of menstrual hygiene management

The percentage distribution of the outcome variables (knowledge, attitude, and practice) presented in Fig. 1 below indicated that 53.45% of respondents had good knowledge of menstrual hygiene management, 70.08% had good attitude towards menstrual hygiene management, while more than half (57.80%) of the respondents practice good menstrual hygiene management including use of disposable sanitary pads, regular hand washing and bathing, and proper disposal of absorbents.

### Sociodemographic characteristics distribution of respondents’ knowledge, attitude, and practice of menstrual hygiene management

#### Sociodemographic characteristics of respondents

As indicated in Table 3, a total of 391 questionnaires were retrieved out of 420, making a response rate of 93.1%. More than half of the respondents (51.41%) were in the age group 12–14 years. About 37% of respondents were in JSS 2. Also, the majority of respondents (62.66%) practice Christianity, while 36.57% are Muslims. Furthermore, a great percentage (76.21%) of respondents’ mothers had at least a secondary level of education and above, while a significant proportion of respondents’ fathers (58.57%) had tertiary education. In addition, 65.98% of respondents lived in a nuclear family structure, while 33.25% lived in an extended family structure. A little above half (51.66%) of the respondents’ parents have more than four children, while only 25.58% of respondents are the first child of their families. More than half of the respondents (53.45%) of the respondents had their first menstruation before the age of 13.

#### Knowledge of menstrual hygiene management

The mean knowledge score was 22.7 ± 3.16, from a maximum obtainable score of 29 (Table 2). Overall, a majority (53.45%) of the respondents had good knowledge of menstruation and menstrual hygiene management, and the main sources of information were the respondents’ mothers (61.85%) and teachers (32.62%) [see SI 1 Table]. Mothers as main sources of information was also evident in the FGD discussion:

*My mummy said if you start menstruation, you will have to be bathing two or three times a day, and you’ll be changing it [sanitary pad] anytime it is full. And anytime you smell odour, go and bathe so that another person will not perceive it* (a participant in JSS Kuje).

In addition, a high proportion (85.17%) of the respondents noted that disposable sanitary pad is the best absorbent for menstruation.

Moreover, almost all the respondents (98.72%) believe that it is appropriate to change menstrual absorbents at least twice a day to maintain good menstrual hygiene. The FGD data also corroborate this result; for instance, one respondent in JSS Pasali noted:

“*the knowledge I had before I started my menstruation was that it is not advisable to use tissue because it is made from a plastic bottle and it has germs so that it can cause infection to your vagina, and I must use three pads [disposable sanitary pad] a day in case there’s heavy flow and I must bath three times a day*” (a participant in JSS Pasali).

Similarly, 96.9% noted that washing hands before and after changing absorbents is essential to maintain menstrual hygiene. Also, a significant proportion (98.21%) of respondents strongly agree or agree that it is necessary to wash hands before and after changing absorbents to maintain good menstrual hygiene (SI 1 Table).

#### Attitude towards menstrual hygiene management

As shown in Table 2, participants (70.08%) had positive attitudes toward menstruation and menstrual hygiene management, with a mean score of 5.0 ± 0.80 with a range of 3 to 6 (Table 2). More than half (55.75) of the respondents stated that menstruation does not affect their association with people, while most (95.65%) respondents attend school during menstruation (SI 2 Table). However, about 2 in 5 participants (44.24%) described their feelings during first menstruation as indifferent as they experienced a combination of feelings. In one of the FGD sessions, a participant noted:

“*When I started my menses, I was not sad, I was happy. Because I was like, wow, finally I reached the woman stage. I’m now a big girl, so I didn’t feel anything sad or embarrassed*” (JSS Area 1).

Again, almost 2 in 5 participants (36.83%) described their response to their first menses as scary, sad, or emotionally disturbing. This is also evident in the FGD sessions:

“*I felt scared because the pain in my belly looked like I wanted to die that day. I was just praying, then I told my mom, and she said I should not worry because I have come of age to see my menstruation*” (a participant in JSS Pasali).

Also, a high proportion (62.15%) of respondents do experience restrictions during their menses, and the major types of restrictions experienced are avoiding prayers (42.80%) and avoiding housework (29.22%).

#### Practice of menstrual hygiene management

On the practice of menstrual hygiene management, the majority of the respondents (57.80%) practice good menstrual hygiene management with a mean score of 31.9 ± 3.6 (Table 2). Almost all (98.21%) of the respondents bathe at least twice daily during their menstruation. According to one FGD participant from JSS Area 1, “*I bathe like twice: in the morning and afternoon*”. On the type of absorbent used during menstruation, disposable sanitary pads were the most used absorbent by respondents (84.91%) while a significant majority (87.21%) expressed their intention to continue using it. The main reasons for continuation, as indicated, are comfortability enjoyed by users (82.70%) and ease of disposal (20.23%) [See SI 3 Table].

“*if I use a pad, I feel comfortable and safe*” - a respondent in JSS Kuje.

In addition, most (98.21%) of the respondents changed their absorbents at least twice per day during menstruation, while only 1.79% changed them once; likewise, almost 98% of the respondents changed their underwear at least twice per day during menstruation. One FGD participant noted:

“*Sometimes I do [change sanitary pad] two times, and sometimes three. Two times I do the morning; when I come back from school, I bathe, and then I put on another one before I sleep. But if the thing is overflowing, I do three times*” (JSS Jabi 1).

In addition, a significant majority (95.91%) of the respondents reported washing their hands both before and after changing their absorbents, indicating good hygiene practices.

Moreover, a majority (87.47%) of the respondents wrapped their used absorbents before disposal. Besides, a significant percentage (72.11%) of the respondents reported receiving support when their menstruation came unexpectedly, such as at school. (See SI 3 Table). This school support is also evident in the FGD sessions:

*“If you need a pad in school, you go to the school clinic, and they will give you a pad to wear*” (JSS Jabi 1).

“*When I see it is full, I take it off, tie it, and run to the counsellor or principal to get extra pads*” (JSS Pasali).

**Table 3:** Sociodemographic characteristics distribution of respondents’ knowledge, attitude, and practice of menstrual hygiene management

Variable	Percentage (%)	Knowledge			Attitude			Practice		Total	
		Good (%)	Poor (%)	*P*-value	Good (%)	Poor (%)	*P*-value	Good (%)	Poor (%)	*P*-value	
**Age Range**											
9–11 years	4.86	31.58	68.42	0.091	68.42	31.58	0.283	57.89	42.11	0.933	19
12–14 years	51.41	51.74	48.26		67.14	32.84		59.20	40.80		201
15–17 years	39.39	56.49	43.51		72.08	27.92		56.49	43.51		154
18 years and above	4.35	70.59	29.41		88.24	11.76		52.94	47.06		17
**Class**											
JSS 1	28.13	40.00	60.00	0.003*	46.36	53.64	0.000*	66.36	33.64	0.079	110
JSS 2	36.57	57.34	42.66		73.43	26.57		52.45	47.55		143
JSS 3	35.29	60.14	39.86		85.51	14.49		56.52	43.48		138
**Religion**											
Christianity	62.66	53.47	46.53	0.898	62.45	37.55	0.000*	64.49	35.51	0.002*	245
Islam	36.57	53.15	46.85		82.52	17.48		46.85	53.15		143
Traditional	0.77	66.67	33.33		100.0	0.0		33.33	66.67		3
**Mother’s level of education**											
No education	10.74	52.38	47.62	0.658	61.90	38.10	0.032*	57.14	42.86	0.263	42
Primary education	13.04	50.98	49.02		76.47	23.53		56.86	43.14		51
Secondary education	34.27	49.25	50.75		62.69	37.31		62.69	37.31		33
Tertiary education	41.94	57.93	42.07		76.22	23.78		54.27	45.73		164
**Father’s level of education**											
No education	8.95	37.14	62.86	0.057	45.71	54.29	0.000*	57.14	42.86	0.402	35
Primary education	5.88	43.48	56.52		78.26	21.74		60.87	39.13		23
Secondary education	26.60	50.00	50.00		60.58	39.42		64.42	35.58		25
Tertiary education	58.57	5852	41.48		77.29	22.71		54.59	45.41		229
**Family Structure**											
Nuclear	65.98	57.36	42.64	0.088	67.44	32.56	0.270	61.63	38.37	0.085	258
Extended	33.25	46.15	53.85		75.38	24.62		50.77	49.23		130
Others	0.77	33.33	66.67		66.67	33.33		33.33	66.67		3
**Age at First Menstruation**											
Below 10 years	4.60	27.78	72.22	0.034*	72.22	27.78	0.857	50.00	50.00	0.776	18
10–12 years	48.85	52.36	47.64		68.59	31.41		59.69	40.31		191
13–15 years	42.97	55.36	44.64		70.83	29.17		57.14	42.86		168
15 years and above	3.58	78.57	21.43		78.57	21.43		50.00	50.00		14
**Parity Level**											
Only child	2.81	45.45	54.55	0.976	81.82	18.18	0.061	63.64	36.36	0.339	11
2 Children	7.42	55.17	44.83		55.17	44.83		68.97	31.03		29
3 Children	17.14	52.24	47.76		61.19	38.81		61.19	38.81		67
4 Children	20.97	52.44	47.56		68.29	31.71		62.20	37.80		82
5 Children above	51.66	54.46	45.54		75.25	24.75		52.97	47.03		202
**Parity Line**											
1st child	25.58	50.00	50.00	0.166	70.00	30.00	0.921	55.00	45.00	0.028*	100
2nd child	23.27	60.44	39.56		68.13	31.87		64.84	35.16		91
3rd child	18.93	52.70	47.30		67.57	32.43		64.86	35.14		74
4th child	14.58	61.40	38.60		73.68	26.32		40.35	59.65		57
Others	17.65	43.48	56.52		72.46	27.54		59.42	40.58		69

*p < 0.05

### Multivariable analysis

#### Demographic factors influencing knowledge, attitude, and practice of respondents on menstrual hygiene management

Table 4 shows the adjusted multivariate regression results of knowledge, attitude, and practice of menstrual hygiene management among JSS adolescent girls in FCT, Nigeria.

The associated factors with knowledge of menstrual hygiene management were class and age at first menstruation. Respondents in JSS 3 [OR = 1.54; 95% CI = 0.84–2.84] and respondents who started their menstruation at age 15 and above [OR = 7.43; 95% CI = 1.26–44.01] were more likely to have good knowledge of menstrual hygiene management than participants who are in JSS2/JSS1 and those who started menstruation before the age of 15.

Factors associated with attitude towards menstrual hygiene management among the JSS adolescent girls in FCT, Nigeria, were class, religion, mothers’ level of education, and father’s level of education. Respondents in JSS 3 [OR = 6.47; 95% CI = 3.34–12.54], respondents who are Muslim [OR = 2.29; 95% CI = 1.63–5.48], and those whose fathers have tertiary education [OR = 3.59; 95% CI = 1.25–10.25], were more likely have a good attitude toward menstrual hygiene management compared to respondents in JSS 2/JSS 1, respondents who practice Christianity/Traditional religion, and those whose fathers have secondary education/no education while respondents whose mother have tertiary education [OR = 0.42; 95% CI = 0.14–1.24] were less likely to have good attitude toward menstrual hygiene compared to respondents whose mothers have secondary/primary/no education.

The associated factors with the practice of menstrual hygiene management among the JSS adolescent girls in FCT, Nigeria were respondents’ religion and parity line. Respondents who practice traditional religion [OR = 0.33; 95% CI = 0.02–4.56] were less likely to practice menstrual hygiene management compared to Christian/Muslim respondents, while respondents who are the third child of their parents [OR = 2.09; 95% CI = 1.04–4.23] were more likely to practice menstrual hygiene compared to respondents whose party lines are 2nd/1st/4th/others.

**Table 4:** Demographic factors influencing knowledge, attitude, and practice of respondents on menstrual hygiene management

Variables	Knowledge			Attitude			Practice		
	OR	CI	*P*-value	OR	CI	*P*-value	OR	CI	*P*-value
**Age group**									
9–11 years	1.00			1.00			1.00		
12–14 years	1.59	0.52–4.92	0.415	0.37	0.10–1.32	0.124	1.12	0.38–3.31	0.833
15–17 years	1.95	0.61–6.27	0.263	0.31	0.08–1.19	0.088	1.22	0.39–3.79	0.726
18–20 years	3.09	0.62–15.41	0.167	0.83	0.09–7.29	0.870	1.45	0.31–6.68	0.631
**Class**	***			***					
JSS 1	1.00			1.00			1.00		
JSS 2	1.49	0.84–2.68	0.171	3.00	1.69–5.32	0.000	0.46	0.25–0.84	0.012
JSS 3	1.54	0.84–2.84	0.164	6.47	3.34–12.54	0.000	0.67	0.35–1.27	0.208
**Religion**				***			***		
Christianity	1.00			1.00			1.00		
Islam	0.93	0.58–1.49	0.767	2.98	1.63–5.48	0.000	0.51	0.31–0.79	0.004
Traditional	0.89	0.06–12.35	0.934	--	--	--	0.33	0.02–4.56	0.370
**Mother’s level of education**				***					
No education	1.00			1.00			1.00		
Primary education	0.67	0.25–1.79	0.422	1.14	0.39–3.27	0.803	0.93	0.35–2.43	0.877
Secondary education	0.49	0.19–1.29	0.153	0.45	0.17–1.21	0.113	1.02	0.41–2.56	0.967
Tertiary education	0.58	0.21–1.63	0.303	0.42	0.14–1.24	0.116	0.85	0.31–2.29	0.743
**Father’s level of education**				***					
No education	1.00			1.00			1.00		
Primary education	1.27	0.37–4.37	0.705	3.69	1.03–13.31	0.045	1.67	0.49–5.69	0.415
Secondary education	1.68	0.59–4.79	0.329	1.81	0.67–4.88	0.244	1.59	0.58–4.35	0.367
Tertiary education	2.51	0.85–7.44	0.096	3.58	1.25–10.25	0.018	1.30	0.46–3.70	0.617
**Family Structure**									
Nuclear	1.00			1.00			1.00		
Extended	0.66	0.41–1.07	0.091	1.48	0.82–2.67	0.192	0.69	0.43–1.12	0.131
Others	0.36	0.03–470	0.439	0.69	0.02–22.00	0.832	0.31	0.02–3.97	0.371
**Age at first menstruation**	***								
Below 10 years	1.00			1.00			1.00		
10–12 years	2.74	0.86–8.74	0.087	0.93	0.27–3.25	0.909	1.58	0.54–4.59	0.400
13–15 years	2.66	0.82–8.56	0.102	0.78	0.21–2.86	0.712	1.49	0.50–4.44	0.467
15 years and above	7.43	1.26–44.01	0.027	0.62	0.09–4.47	0.634	1.00	0.21–4.68	1.000
**Parity Level**									
Only child	1.00			1.00			1.00		
2 Children	1.74	0.39–7.77	0.463	0.38	0.06–2.54	0.320	0.96	0.21–4.45	0.956
3 Children	1.56	0.38–6.39	0.540	0.44	0.07–2.69	0.374	0.62	0.15–2.59	0.508
4 Children	1.42	0.35–5.80	0.624	0.64	0.10–3.95	0.632	0.94	0.22–3.92	0.927
5 Children above	1.89	0.48–7.51	0.366	0.99	0.16–5.94	0.987	0.59	0.14–2.39	0.459
**Parity line**							***		
1st child	1.00			1.00			1.00		
2nd child	1.41	0.75–2.67	0.287	0.72	0.34–1.52	0.391	1.82	0.95–3.51	0.017
3rd child	1.10	0.56–2.17	0.775	0.81	0.36–1.80	0.602	2.09	1.04–4.23	0.039
4th child	1.38	0.65–2.93	0.395	0.75	0.30–1.87	0.540	0.59	0.28–1.28	0.187
Others	0.68	0.32–1.40	0.295	0.60	0.25–1.47	0.266	1.79	0.86–3.76	0.121

***p < 0.05

## Discussion

This study examined knowledge, attitude, and practice of menstrual hygiene management among JSS adolescent girls in the FCT, Nigeria, using a mixed method of quantitative and qualitative data collection and analysis. The findings contributed to the existing literature and offer valuable insights on various sociodemographic factors influencing menstrual hygiene management among JSS adolescent girls, thereby providing valuable insights for policymakers, educators, and health practitioners aiming to improve menstrual hygiene practices and overall adolescent health in the region. Understanding the sociodemographic characteristics influencing knowledge, attitude, and practice toward menstrual hygiene management is crucial for designing effective interventions. These findings align with previous studies emphasising the importance of education and awareness in fostering positive menstrual hygiene management among school adolescents [17, 25, 26].

The results of this study indicate a complex outlook regarding knowledge of menstrual hygiene management among JSS adolescent girls in the FCT, with half of the participants (53.45%) demonstrating good knowledge. This is similar to findings from other studies in Nigeria [25, 27, 28] and other countries [29–31]. The good knowledge of menstruation and menstrual hygiene management exhibited by the respondents may be attributed to the high level of awareness and interventions of menstrual hygiene management among adolescents within Nigeria and other developing countries.

It is noteworthy that the data collection for this study was conducted as a part of the 2023 edition of the “Pad-A-Girl” Project, which was organised to commemorate the annual United Nations Menstrual Hygiene Day. The primary objective of this project was to deliver comprehensive health education on menstrual hygiene and distribute disposable sanitary pads to junior secondary school (JSS) female adolescents in the FCT. It is believed that the students had previously benefited from similar interventions, alongside health education received both in schools and at home [32]. Hence, this collective exposure likely contributed to the good knowledge of menstrual hygiene exhibited by the respondents. However, concerns remain regarding the other half of the participants who demonstrated poor knowledge of menstruation and menstrual hygiene management.

Factors associated with knowledge of menstrual hygiene management were respondents’ class and age at first menstruation. The respondents in JSS 3 and those who experienced menarche at an older age (15 years and above) exhibited better knowledge of menstrual hygiene management. This is a variant of reports from other similar studies in Nigeria [25, 28]^,^ Ghana [29, 30] and Ethiopia [33] in which respondents’ age, level of income of parents, level of education of parents, and place of residents were associated with knowledge of menstrual hygiene among respondents. This suggests the potential role of age and educational level in shaping awareness and understanding of menstrual health among adolescent girls.

The results on attitudes toward menstrual hygiene management showed that the majority of the respondents (70.08%) exhibited positive attitudes towards menstruation and menstrual hygiene management, suggesting a relatively favourable perception among this demographic. This result is consistent with previous studies [34, 35], which also reported predominantly positive attitudes towards menstruation among adolescent girls. However, it is noteworthy that 3 in 5 of the participants (62.15%) reported experiencing restrictions during menstruation. This supports the findings of previous studies carried out in Nigeria [25, 34], Ghana [30], Somalia [36], and Bangladesh [37], which documented the prevalence of menstruation-related stigma restrictions among adolescent girls.

The finding on the issue of restriction may be associated with several factors including the cultural, religious, and environment of the respondents. This reflects the existence of menstrual taboos and cultural beliefs surrounding menstruation in Nigeria and other countries, which often dictate restrictive practices during menstruation [38]. Addressing these cultural norms and taboos surrounding menstruation is crucial for promoting the rights and well-being of adolescent girls and ensuring their access to comprehensive menstrual hygiene management practices. Efforts to challenge and transform these beliefs through targeted education and advocacy interventions are imperative to empower adolescent girls to manage their menstruation with dignity and autonomy.

The findings on attitude towards menstrual hygiene management further showed that the respondents who are in the JSS 3 class, Muslim, and those whose fathers had tertiary education were more likely to have positive attitudes toward menstrual hygiene management compared to their colleagues, indicating the influence of socio-cultural factors on perceptions and beliefs surrounding menstruation. This study is in line with a previous study [25] that concluded adolescents’ attitudes towards menstruation and menstrual hygiene management are influenced by their sociodemographic factors, including religious belief, level of education, and parental level of education, among others.

This underscores the significance of socio-cultural factors in shaping perceptions and beliefs surrounding menstruation. Tailored interventions that consider diverse cultural norms and family dynamics are essential for promoting optimal menstrual hygiene practices among adolescent girls.

On the practice of menstrual hygiene management, overall, more than half of the respondents (57.80%) of the respondents practice good menstrual hygienic. This finding is consistent with similar studies in Nigeria [26, 34, 39], Ghana [29] and Ethiopia [40], where the majority of the respondents practice good menstrual hygiene management. The study indicates that respondents who demonstrated a good understanding of menstruation and menstrual hygiene also engaged in good menstrual hygiene management.

The practice of menstrual hygiene management was influenced by factors such as religion and parity level, underscoring the need for tailored interventions addressing diverse cultural norms and family dynamics. The result shows that respondents practising traditional religion were less likely to practice menstrual hygiene management compared to Christian/Muslim respondents, emphasising the importance of religion-sensitive approaches in promoting optimal hygiene behaviours. This is in line with the finding of a study conducted in Ghana by Asumah et al. [10] that reported that religion and culture have very dire consequences on effective menstrual hygiene management. In variance, respondents with specific birth order positions (3rd child) are more likely to practice menstrual hygiene management compared to 2nd/1st/4th child/others. The finding that respondents who are the third child of their parents are more likely to practice menstrual hygiene compared to those who fall into other birth order categories is intriguing and suggests a potential link between birth order and menstrual hygiene behaviour. This requires further study to better understand the underlying factors influencing menstrual hygiene management practice in relation to birth order.

### Strengths and limitations

Findings from this study should be interpreted in the light of some limitations. First, the study’s cross-sectional design limits the ability to establish causal relationships between variables. As data was collected at a single point in time, it is challenging to discern the temporal sequence of events and draw definitive conclusions about cause and effect. Also, the findings may not be fully generalisable to other regions or populations beyond junior secondary school adolescent girls in the FCT, Nigeria. This is because factors such as cultural variations and socio-economic differences could influence the results in different contexts. Moreover, while the study acknowledges emotional challenges during the first menstruation, it does not delve deeply into the nature and impact of these challenges. Therefore, future research could explore this aspect more comprehensively to inform targeted interventions.

Nevertheless, the study utilised a cross-sectional mixed-method design, combining quantitative surveys and FGDs. This approach allowed for a more holistic understanding of the knowledge, attitude and practice of MHM among adolescent girls. Also, the study included a diverse sample of 420 participants from four government junior secondary schools, chosen through a multistage sampling technique. This diversity enhances the generalizability of the findings to a broader population. In addition, the qualitative findings were consistent with the quantitative findings across the knowledge, attitude, and practice variables. This triangulation of data sources strengthens the reliability of the study’s conclusions. Besides, the conclusion and recommendations highlight practical steps for improving MHM, including school-based interventions, parental and community involvement, mental health support, hygiene facilities, curriculum integration, peer education programs, and community sensitisation. These recommendations provide a roadmap for implementing effective interventions.

### Practical implications and future studies

The findings of this study underscore the influence of sociodemographic factors on knowledge, attitude and practice of menstrual hygiene management among JSS adolescent girls in the FCT, Nigeria. Practical implications of the findings suggest the importance of tailored interventions in promoting menstrual hygiene management among junior secondary school adolescent girls. Firstly, considering the influence of sociodemographic factors such as class, religion, parental education, and birth order on knowledge, attitude, and practice of menstrual hygiene management, policymakers and educators must develop culturally sensitive and context-specific educational programs. These programs should address not only the biological aspects of menstruation but also the socio-cultural beliefs and practices surrounding menstruation that may impact girls’ attitudes and behaviours. In addition, efforts to increase access to menstrual hygiene products, such as disposable sanitary pads, and to create supportive environments in schools and communities are essential. This may include ensuring the availability of sanitary facilities, providing comprehensive menstrual health education, and fostering open discussions to challenge menstrual taboos and stigma.

Furthermore, future studies should delve deeper into understanding the underlying factors contributing to variations in menstrual hygiene practices among adolescent girls, including the influence of birth order. Longitudinal studies could explore how family dynamics and birth order influence girls’ perceptions and behaviours related to menstruation over time. Additionally, qualitative research could provide insights into the cultural norms and societal expectations surrounding menstruation within different family structures. Moreover, interventions targeting specific subgroups, such as girls from traditional religious backgrounds or those with different birth order positions, should be developed and evaluated to address their unique needs and challenges in menstrual hygiene management. Ultimately, advancing knowledge in this area will inform the design of more effective interventions and policies to promote menstrual health and well-being among adolescent girls in Nigeria and beyond.

## Conclusion

The study concluded that the majority of respondents demonstrated good knowledge, positive attitudes, and favourable practices towards menstrual hygiene management. Moreover, our findings highlight the influence of sociodemographic factors such as class, age at first menstruation, religion, and parental education on these outcomes. Importantly, while our study reveals encouraging trends in the knowledge, attitude and practice of menstrual hygiene management, it also underscores the need for targeted interventions to address cultural norms, religious beliefs, and family dynamics that may impact the attitude and practice of menstrual hygiene. By implementing comprehensive health education programs, ensuring access to affordable menstrual hygiene products, and challenging restrictive cultural taboos, policymakers, educators, and health practitioners can play a crucial role in promoting optimal menstrual hygiene management among adolescent girls, thus safeguarding their reproductive health and well-being in Nigeria and beyond.

### Electronic supplementary material

Below is the link to the electronic supplementary material.

Supplementary Material 1

Supplementary Material 2

## Data Availability

The data that support the findings of this study are available from the FCT Health Research Ethics Committee, but restrictions apply to the availability of these data, which were used under licence for the current study and so are not publicly available. The data are, however, available from the authors upon reasonable request and with the permission of the FCT Health Research Ethics Committee. The questionnaire used to collect information from the respondents has been included as a supplementary file.

## Abbreviations

FCT: Federal Capital Territory
FGDs: Focus Group Discussions
IDP: Internally Displaced Person
JMP: Joint Monitoring Programme
JSS: Junior Secondary Schools
KAP: Knowledge, Attitude, and Practice
LMICs: Low- and Middle-Income Countries
MHM: Menstrual Hygiene Management
NGOs: Non-Governmental Organisations
PDF: Preston Development Foundation
RAND: Random
SDGs: Sustainable Development Goals
UBE: Universal Basic Education
UBEB: Universal Basic Education Board
UN: United Nations
UNICEF: United Nations Children’s Fund
WASH: Water Sanitation and Hygiene
WHO: World Health Organization
