# Supplementary material for: “If I use pad, I feel comfortable and safe”: a mixed-method analysis of knowledge, attitude, and practice of menstrual hygiene management among in-school adolescent girls in a Nigerian city
Source: BMC Public Health. 2024 Jun 27;24:1721. doi: 10.1186/s12889-024-19256-5 (PMC11212270; doi:10.1186/s12889-024-19256-5)
Supplement: Supplementary file 1 — Supplementary Material 1 [file 12889_2024_19256_MOESM1_ESM.docx]

**Supporting Information (SI)**

### **SI 1 Table: Descriptive statistics on knowledge of menstruation and menstrual hygiene management among JSS adolescent girls in FCT, Nigeria.**

|  | Variable | Frequency | Percentage (%) |
| --- | --- | --- | --- |
| 1 | **Did you hear about menstruation before your first experience?** |  |  |
| I | No | 36 | 9.21 |
| Ii | Yes | 355 | 90.79 |
|  | **Total** | **391** | **100.00** |
| 1b | **Source of information (first hearing)** | **(N=355)** |  |
| i | Mother | 214 | 60.28 |
| ii | Elder sister | 77 | 21.69 |
| iii | Friend | 32 | 9.01 |
| iv | Teacher | 101 | 28.45 |
| v | Relatives | 38 | 10.70 |
| vi | Media | 5 | 1.41 |
| vii | Others | 4 | 1.13 |
| 2. | **Did you have any prior knowledge of menstrual hygiene management before your first experience?** |  |  |
| I | No | 66 | 16.88 |
| Ii | Yes | 325 | 83.12 |
|  | **Total** | **391** | **100.0** |
| 2b | **Source of information (prior knowledge)** | **(N=325)** |  |
| i | Mother | 201 | 61.85 |
| ii | Elder sister | 58 | 17.85 |
| iii | Friend | 27 | 8.31 |
| iv | Teacher | 106 | 32.62 |
| v | Relatives | 25 | 7.69 |
| vi | Media | 4 | 1.23 |
| vii | Others | 8 | 2.46 |
| 3 | **Is menstruation a normal process in females** |  |  |
| I | No | 17 | 4.35 |
| Ii | Yes | 374 | 95.65 |
|  | **Total** | **391** | **100.00** |
| 4 | **Do you think poor menstrual hygiene can result in infection** |  |  |
| I | No | 33 | 8.44 |
| Ii | Yes | 358 | 91.56 |
|  | **Total** | **391** | **100.00** |
| 5 | **Which of the absorbent material is best used for menstruation?** |  |  |
| I | Cotton wool | 5 | 1.28 |
| Ii | Menstruation cup | 3 | 0.77 |
| Iii | Old piece of cloth | 9 | 2.30 |
| Iv | Piece of cloth | 16 | 4.09 |
| v. | Reusable sanitary pad | 16 | 4.09 |
| vi. | Sanitary pad | 333 | 85.17 |
| vii. | Tampons | 1 | 0.26 |
| viii. | Tissue/Toilet paper | 5 | 1.28 |
| ix. | Others | 3 | 0.77 |
|  | **Total** | **93** | **100.00** |
| 6 | **How many times do you think is appropriate to change your menstrual absorbent daily?** |  |  |
| i | No need | 1 | 0.26 |
| ii | Once | 4 | 1.02 |
| iii | Twice | 96 | 24.55 |
| iv | More than twice | 290 | 74.17 |
|  | **Total** | **391** | **100.00** |
| 7 | **It is necessary to wash hands before and after changing absorbent to maintain good menstrual hygiene** |  |  |
| I | Agree | 114 | 29.16 |
| Ii | Strongly agree | 270 | 69.05 |
| Iii | Indifference | 4 | 1.02 |
| iv | Strongly Disagree | 1 | 0.26 |
| v | Disagree | 2 | 0.51 |
|  | **Total** | **391** | **100.00** |
|  |  |  |  |
| 8 | **How many times is it appropriate to bath in a day during the menstruation period** |  |  |
| I | No need | 1 | 0.26 |
| Ii | Once | 4 | 1.02 |
|  | Twice | 108 | 27.62 |
|  | More than twice | 278 | 71.10 |
|  | **Total** | **391** | **100.00** |
| 9 | **Record Knowledge** |  |  |
|  | Poor Knowledge | 182 | 46.55 |
|  | Good Knowledge | 209 | 53.45 |
|  | **Total** | **391** | **100.00** |

**SI 2 Table: Descriptive Statistics on attitude towards menstruation and menstrual hygiene management among JSS adolescent girls in FCT, Nigeria**

|  | Variable | Frequency | Percentage (%) |
| --- | --- | --- | --- |
| 1 | **How did you feel during your first menstruation?** |  |  |
| i | Emotionally Disturbed | 72 | 18.41 |
| ii | Happy | 74 | 18.93 |
| iii | Indifference | 173 | 44.24 |
| iv | Sad | 20 | 5.12 |
| v | Scared | 52 | 13.30 |
|  | **Total** | **391** | **100.00** |
| 2 | **Do you experience restrictions on normal activities during menstruation?** |  |  |
| i | No | 148 | 37.85 |
| ii | Yes | 243 | 62.15 |
|  | **Total** | **391** | **100.00** |
| 2b | **What type of restriction** | **(N=243)** |  |
| i | Avoid celebrations and festivals | 45 | 18.52 |
| ii | Avoid prayer | 104 | 42.80 |
| iii | Avoid housework | 71 | 29.22 |
| iv | Others | 40 | 16.46 |
| 3 | **Does menstruation affect your association with people?** |  |  |
| i | No | 218 | 55.75 |
| ii | Yes | 173 | 44.25 |
|  | **Total** | **391** | **100.00** |
| 3b | **Why** | **(N=173)** |  |
| i | Fear of unexpected bleeding/pain | 110 | 63.58 |
| ii | Fear of odour | 48 | 27.75 |
| iii | Presence of menstrual symptoms | 34 | 19.65 |
| iv | Others | 11 | 6.36 |
| 4 | **Do you attend school during menstruation?** |  |  |
| i | No | 17 | 4.35 |
| ii | Yes | 374 | 95.65 |
|  | **Total** | **96** | **100.00** |
| 4b | In No, why | (N=17) |  |
| i | Lack of toilet facility | 10 | 58.82 |
| ii | Lack of water | 1 | 5.88 |
| iii | Lack of disposal facility | 4 | 23.53 |
| iv | Fear of unexpected bleeding/stain | 4 | 23.53 |
| v | Presence of menstrual symptoms | 3 | 17.65 |
| vi | Lack of absorbent | 0 | 00.0 |
| vii | Others | 2 | 11.76 |
| 5 | **Record of Attitude** |  |  |
|  | Poor Attitude | 117 | 29.92 |
|  | Good Attitude | 274 | 70.08 |
|  | **Total** | **391** | **100.00** |

**SI 3 Table: Descriptive statistics on the practice of menstrual hygiene management among JSS adolescent girls in FCT, Nigeria.**

|  | Variable | Frequency | Percentage (%) |
| --- | --- | --- | --- |
| 1 | **How many times do you bathe per day during menstruation?** |  |  |
| i | More than thrice | 86 | 21.99 |
| ii | Thrice | 180 | 46.04 |
| iii | Twice | 118 | 30.18 |
| iv | Once | 7 | 1.79 |
|  | **Total** | **391** | **100.00** |
| 2 | **What type of absorbent do you mostly use during your menstruation period?** |  |  |
| i | Cotton wool | 4 | 1.02 |
| ii | Menstruation cup | 1 | 0.26 |
| iii | Old piece of cloth | 7 | 1.79 |
| iv | Piece of cloth | 27 | 6.91 |
| v | Reusable sanitary pad | 9 | 2.30 |
| vi | Sanitary pad | 332 | 84.91 |
| vii | Tissue/Toilet paper | 7 | 1.79 |
| viii | Others | 4 | 1.02 |
|  | **Total** | **391** | **100.00** |
| 3 | **Would you like to continue using the current absorbent you are using for your menstruation?** |  |  |
| i | No | 50 | 12.79 |
| ii | Yes | 341 | 87.21 |
|  | **Total** | **391** | **100.00** |
| 3b | **If Yes, why** | **(N=341)** |  |
| i | It is comfortable | 282 | 82.70 |
| ii | It is cheap | 43 | 12.61 |
| iii | It is reusable | 13 | 3.81 |
| iv | It is easy to dispose | 69 | 20.23 |
| v | Others | 24 | 7.04 |
| 4 | **How many times do you change your absorbent per day during your menstruation?** |  |  |
| i | Once | 7 | 1.79 |
| ii | Twice | 93 | 23.79 |
| iii | Thrice | 209 | 53.45 |
| iv | More than thrice | 82 | 20.97 |
|  | **Total** | **391** | **100.00** |
| 5 | **How many times do you change your underwear per day during your menstruation?** |  |  |
| i | Never | 1 | 0.26 |
| ii | Once | 8 | 2.05 |
| iii | Twice | 111 | 28.39 |
| iv | Thrice | 198 | 50.64 |
| v | More than thrice | 73 | 18.67 |
|  | **Total** | **391** | **100.00** |
| 6 | Do you wash your hands before and after changing your absorbent? |  |  |
| i | No | 16 | 4.09 |
| iv | Yes | 375 | 95.91 |
|  | **Total** | **93** | **100.00** |
| 7 | **Do you clean your genitalia during menstruation?** |  |  |
| i | No | 4 | 1.02 |
| iv | Yes | 387 | 98.98 |
|  | **Total** | **391** | **100.00** |
| 8 | **How do you mostly dispose of used absorbents?** |  |  |
| i | Burning | 3 | 0.77 |
| ii | Burying | 25 | 6.39 |
| iii | Dustbin | 5 | 1.28 |
| iv | Latrine/toilet | 81 | 20.72 |
| v | Others | 277 | 70.84 |
|  | **Total** | **391** | **100.00** |
| 9 | **Do you wrap your absorbent before disposal?** |  |  |
| i | No | 49 | 12.53 |
| ii | Yes | 342 | 87.47 |
|  | **Total** | **391** | **100.00** |
| 9b | **Type of absorbent wrapper** | **(N=342)** |  |
| i | Paper | 23 | 6.73 |
| ii | Used cloth | 25 | 7.31 |
| iii | Plastic bag | 259 | 75.73 |
| iv | Others | 35 | 10.23 |
| 10 | **In the event that your menstruation comes unexpectedly (e.g., at school), do you receive any support?** |  |  |
| i | No | 106 | 27.89 |
| ii | Yes | 274 | 72.11 |
|  | **Total** | **391** | **100.00** |
| 11 | **What do you do to prevent menstrual pain?** |  |  |
| i | Avoid eating sugary food | 135 | 34.53 |
| ii | Exercise | 23 | 5.88 |
| iii | Nothing | 117 | 29.92 |
| iv | Others | 22 | 5.63 |
| v | Take more fruits | 8 | 2.05 |
| vi | Take pain relief medication | 86 | 21.99 |
|  | **Total** | **391** | **100.00** |
| 12 | **Record of Practice** |  |  |
| i | Poor practice | 165 | 42.20 |
| ii | Good practice | 226 | 57.80 |
|  | **Total** | **391** | **100.0** |
