# Supplementary material for: “If I use pad, I feel comfortable and safe”: a mixed-method analysis of knowledge, attitude, and practice of menstrual hygiene management among in-school adolescent girls in a Nigerian city
Source: BMC Public Health. 2024 Jun 27;24:1721. doi: 10.1186/s12889-024-19256-5 (PMC11212270; doi:10.1186/s12889-024-19256-5)
Supplement: Supplementary file 2 — Supplementary Material 2 [file 12889_2024_19256_MOESM2_ESM.pdf]

QUESTIONNAIRE ON KNOWLEDGE, ATTITUDE, AND PRACTICE OF MENSTRUAL HYGIENE MANAGEMENT AMONG JUNIOR SECONDARY SCHOOL ADOLESCENT GIRLS IN THE FEDERAL CAPITAL TERRITORY,NIGERIA

Dear Respondent,

This questionnaire is designed to elicit information on the knowledge, attitude, and practice of menstrual hygiene management among Junior Secondary School adolescent girls in the Federal Capital Territory, Nigeria. All information provided will be used solely for the purpose of thisresearch and will be treated with the utmost confidentiality. Your honest and objective response to the questions will be highly appreciated.

Thanks for your anticipated cooperation.

BACKGROUND INFORMATION (This is to be filled by the data collectors)

1. Name of School: ____
2. Location of School: Rural ( ) Urban ( )

SECTION A: DEMOGRAPHIC INFORMATION

Instruction: Kindly tick (√) in the box as appropriate for each of the following items.

1) **Age range:**
   (a) 9 - 11 years old ( ) (b) 12 - 14 years old ( ) (c) 15 - 17 years old ( )
   (d) 18 – 20 years old ( ) (e) 21 years and above ( )

2) **Class:**
   (a) JSS 1 ( ) (b) JSS 2 ( ) (c) JSS 3 ( )

3) **Religion:**
   (a) Christianity ( ) (b) Islam ( ) (c) Traditional ( ) Others: Please specify ______________________

4) **Ethnicity:**
   (a) Gbagyi ( ) (b) Amwamwa ( ) (c) Bassa ( ) (d) Egbira ( ) (e) Gade ( )
   (f) Ganagana ( ) (g) Gbari ( ) (h) Gwandara ( ) (i) Koro ( ) (j) Hausa ( ) (k) Igbo ( )
   (l) Yoruba ( ) (m) Others: Please specify ___________________________________

5) **Location of residence:** (a) Urban ( ) (b) Rural ( )

6) **Mother's Highest Level of Education:**
(a) No education ( ) (b) Primary education ( ) (c) Secondary education ( ) (d) Tertiary Education ( )

7) **Father's Level of Education:**
(a) No education ( ) (b) Primary education ( ) (c) Secondary education ( ) (d) Tertiary Education ( )

8) **Family Structure:**
(a) Nuclear ( ) (b) Extended ( ) Others: Please specify ----------------

9) **Age at first Menstruation:**
(a) bellow 10 ( ) (b) 10 - 12 ( ) (c) 13 - 15 ( ) (d) 15 and above ( )

10) What is the parity level? (a) only child (b) 2 children (c) 3 children (d) 4 children (e) 5 childrenand above

11) What is your parity line in the family? (a) 1st child (b) 2nd child (c) 3rd child (d) 4th child (e) lastchild

SECTION B: KNOWLEDGE OF MENSTRUAL HYGIENE MANAGEMENT

1. Did you hear about menstruation before your first experience? a.) Yes ( ); b.) No ( )

1b: What was the source of information: a.) Mother ( ); b.) Elder sister ( ); c.) Friend ( ); d.) Teacher ( ); e.) Relatives ( ); f.) Media ( ); (g) Others: Please specify ___________________

2. Did you have any prior knowledge of menstrual hygiene management before your first menstruation? a.) Yes ( ); b.) No ( )

2b: What was the source of information: a.) Mother ( ); b.) Elder sister ( ); c.) Friend ( ); d.) Teacher ( ); e.) Relatives ( ); f.) Media ( ); (g) Others: Please specify ___________________

3. Is menstruation a normal process in females? a.) Yes ( ); b.) No ( )

4. Do you think poor menstrual hygiene can result in infection? a.) Yes ( ); b.) No ( )

5. Good Menstrual Hygiene Management can prevent menstrual pain? a.) Yes ( ); b.) No ( )

6. Which of the absorbent materials is best used for menstruation?
a.) Cotton wool ( ); b.) Tissue/Toilet paper ( ); c.) Old piece of cloth ( ); d.) New pieceof cloth ( ); e.) Sanitary pad ( ); f.) Menstruation cup ( ); g.) Reusable sanitary pad ( ); h.) Others: Please specify ___

7. How many times do you think is appropriate to change menstrual absorbent daily?

(a) Once (b) twice (c) more than twice (d) no need

8. It is needful to wash hands before and after changing absorbent to maintain good menstrualhygiene.
(a.) Strongly agreed (b) Agree (c) Indifference (d) Disagree (e) Strongly disagree

9. How many times is it appropriate to bathe in a day during the menstruation period?
a.) Once ( ); b.) Twice (c) more than twice (d) No need

SECTION C: ATTITUDE TOWARD Menstruation and MENSTRUAL HYGIENEMANAGEMENT

1) How did you feel during your first menstruation?
a.) Happy ( ); b.) Sad ( ); c.) Scared ( ); d.) Emotional disturbed ( ); (e) Indifference
(f).) Others: Please specify ___________________

2) Do you experience restriction of normal activities during menstruation?a.) Yes ( ); b.) No ( )

2b. What type of restriction?
a.) Avoid celebrations and festivals ( ); b.) Avoid prayer ( ); c.) Avoid housework ( ) d.) Others: Please specify _________________________

3) Does menstruation affect your association with people? a.) Yes ( ); b.) No ( )

3b) If yes, why? a.) Fear of unexpected bleeding/stain b.) Fear of odor c.) Presence ofmenstrual symptoms d.) Others: Please specify _____________________________________

4) Do you attend school during menstruation? a.) Yes ( ); b.) No ( )
If No, why a.) Lack of toilet facility ( ); b.) Lack of water ( ); c.) Lack of disposal facility ( ); d.) Fear of unexpected bleeding/stain ( ) e.) Presence of menstrual symptoms ( ); f.) Lack of absorbent ( ); g.) Others: Please specify _________________________

SECTION D: PRACTICE OF MENSTRUAL HYGIENE MANAGEMENT

1) How many times do you bath per day during menstruation?
a.) Never ( ); b.) Once ( ); c.) Twice ( ); d.) More than twice ( )

2) What type of absorbent do you mostly use during your menstruation period?

PRESTON DEVELOPMENT FOUNDATION
PDF

a.) Cotton wool ( ); b.) Tissue/Toilet paper ( ); c.) Old piece of cloth ( ); d.) New piece of cloth ( ); e.) Sanitary pad ( ); f.) Menstruation cup ( ); g.) Reusable sanitary pad ( ); h.) Others : Please specify ____

3) Do you like to continue using the current absorbent you are using for your mensuration? a.)Yes ( ); b.) No ( )

3b) If yes, why? Tick as many as possible a.) It is comfortable ( ); b.) It is cheap ( ); c.) It is reusable ( ); d.) It is easy to disposed of ( ) e.) Others: Please specify ____________________

3c) If no, why? Tick as many as possible a.) It is not comfortable ( ); b.) It is not cheap ( ); c.) It is not reusable ( ); d.) It is not easy to dispose ( ) e.) Others: Please specify _____________________

4) How many times do you change your absorbent per day during menstruation?
a.) Never ( ); b.) Once ( ); c.) Twice ( ); d.) Thrice ( ); e.) More than thrice ( )

5) How many times do you change your underwear per day during menstruation?
a.) Never ( ); b.) Once ( ); c.) Twice ( ); d.) Thrice ( ); e.) More than thrice ( )

6) Do you wash your hands before and after changing your absorbent? a.) Yes ( ); b.) No ( )
7) Do you clean your genitalia during menstruation? a.) Yes ( ); b.) No ( )
8) How do you dispose used absorbent? a.) Dustbin ( ); b.) Open dumping ( ); c.) Burning( ); d.) Latrine/toilet ( ); e.) Burying ( ). f.) Others: Please specify ______________________________

9) Do you wrap your used absorbent before disposal? a.) Yes ( ); b.) No ( )
If yes, what type of absorbent wrap do you mostly use for disposing used absorbent? a.) paper( ); b.) used cloth ( ); c.) Plastic bag ( ); d.) Others: Please specify ______________________________

10) In the event that your menstruation comes unexpectedly (e.g., school), do you receive anysupport?
Yes ( ) No ( )

11) If Yes, from who ______________________________

12) If yes, what type of support? Provision of materials ( ) Others

13) What do you do to prevent menstrual pain?
a.) Nothing ( ); b.) Exercise ( ); c.) Take pain relief medication ( ); d.) Avoid eating sugary food ( ); e.) Take more fruits ( ). f.) Others: Please specify ______________________________

FGD GUIDE

KNOWLEDGE, ATTITUDE, AND PRACTICE OF MENSTRUAL HYGIENE MANAGEMENT AMONG JUNIOR SECONDARY SCHOOL ADOLESCENT GIRLS IN THE FEDERAL CAPITAL TERRITORY, NIGERIA

Dear Discussants,

This questionnaire is designed to elicit information on the knowledge, attitude and practice of menstrual hygiene management among Junior Secondary School adolescent girls in the Federal Capital Territory, Nigeria. All information provided will be used solely for the purpose of this research and will be treated with the utmost confidentiality. Your honest and objective response to the questions will be highly appreciated.

Note to Moderator:

Before turning on the recorder:

- Introduce yourself and other team members
- Go over the information leaflet and consent form
- Go over broadly what the interview will be about
- Assure them that there is no wrong answer, everything they say will be helpful – we are interested in knowing their views and it is in no way testing or judging them
- Assure them that everything they say will be strictly confidential and will only be shared with people involved in the study.
- The data collected will be anonymized so others will not be able to identify them.
- Explain you may note things down as you go along, so that you can revisit these later
- There is be informal conversation prior to the commencement of discussion to build rapport and trust between you and the participant. Therefore, talk to them as you would talk to a friend until you feel they are comfortable e.g, ask how their day was, what sports they play in school….etc
- Assign numbers to each respondent for anonymity purposes, and ensure that there are no chorus answers. That is, set up ground rules that inform them that anybody that wants to answer should raise up their hands.

During the interview

- Once you start the interview; if you feel the participant is holding back for some questions, remind them again about confidentiality of answers and that there is no wrong answer.
- If the participant doesn't answer the question, give them some time to collect their thoughts and respond, but if you still do not get a response, check that they understand what you mean, ask them to repeat the question back to you to verify.
- If the participant says something interesting and relevant to the topic, ask them more about it, dig deeper into the area (even though it is not on the guide).

Focus Group Discussion Guide (post-menarche girls)
**Ice breaker**
**1. First, we would like to start with introductions. Can everyone introduce herself and tell us:**<br>• *What grade you are in?*<br>• *Your favorite subject in school*<br>• *Favorite color*<br>• *Favorite food etc. (continue till the girls start responding more openly)*<br><br>**2. What do you enjoy the most about school?**
**Key Questions**
**3. Can you all describe the usual condition of the toilet facilities at this school?**<br>Probe:<br>• How many are there?<br>• Are there separate toilets for boys and girls?<br>• Comment on: cleanliness? smell? privacy? safety?<br>• Availability of soap/water/materials for personal hygiene?<br>• Do girls prefer going with friends?<br>• How do girls dispose used absorbents?<br>**4. Can you explain any school rules regarding toilet use?**<br>Probe:<br>• Do all students use the toilets?<br>• Is there any other place, asides the toilet used for waste excretion? Where? Why?<br>• Is there a specific time for using the toilets? (e.g., only during break, at any point needed?) When can students use toilets?)<br>• Are the toilets ever locked? How accessible are they? (are there any times a girl cannot use the toilet?)<br>• Is water readily available? Either well, running tap water, etc.<br>• If hauling water is needed, who does this?<br>*Hint: “mairuwa” or any water that is being transported to the school*<br>• Who cleans the toilets?

Thank you. I would now like to discuss menstruation practices.

5a. What do girls in this school call menstruation?

Are there any 'secret' words or terms or slang used so nobody else will know what is discussed? If any, what do these terms mean? Negative or positive connotation?

5b. When you hear these slangs, how does it make you feel?

5c. What do you think about menstrual blood?

6. When do you think most girls learn about menstruation? And from whom?
(*Personalize the question to the participants if they did not respond accurately).*

7. What knowledge did you have about menstruation before you saw your first menstruation?
Probe:

- Were there any stories told by grandmothers or mothers or elder female in the family?
- Why do women menstruate?
- Do women menstruate when they are pregnant?
- What is menstrual blood and where does it come from?
- Is there anything women are not allowed to do while they menstruate?

8a. Did you feel prepared for your first menstruation? Why?
Hint: menarche means the first course of menstruation

8b. How did you react when you saw your menstruation for the first time?
(*Personalize the question to the participants if they did not respond accurately).*
Probe: Do they feel happy, scared, emotionally disturbed, etc.

9. What type of absorbent do you think that the girls in this school use mostly during menstruation?
Probe:

- Why do you think that is the most commonly used absorbent? What is the cost? *(try establish if there are non-monetary costs, e.g., maybe their parent/guardian purchases it for them)*
- How accessible are they? (Is it difficult to get? Where do you get it from?)

10. Are there any activities that girls who are menstruating are excluded from? (e.g., prayers, cooking, school, etc.)

- *Do these restrictions interfere with school tasks??*
- *How do you feel about these restrictions?*
- *Do these restrictions cause some girls to be absent from school for some days?*
- *Do you think that mensuration affects a girl's academic performance in school? Why?*

11. Can you walk me through the process of changing an absorbent material? That is what are the steps I need to take if I am menstruating and I want to change my absorbent material?
Probe:

- *How many times in a day do you change your absorbent material when you are menstruating?*
- *If you need to change your absorbent material during menstruation, where do you change it? (List as many as possible)*
- *Why do you change in that place? (probe for safety, cleanliness, privacy, etc.)*

12. If you want to dispose a used absorbent material, where will you dispose of it?

13. How are practices of *young girls* different to those of women during menstruation? (That is, what do you think is different about how you and your parent/ward/aunty handle menstruation? Is there something that you do that they don't do? Or vice versa?)

Closing Questions
**Thank you. We just have a few more questions.**<br><br>**14. What are some of the things you worry about when going to school during your menstruation?**<br><br>**15. For the girls whose school attendance is affected during their menstruation, what do you think can help improve their school attendance during menstruation?**<br><br>**16. If boys knew you were menstruating, do you think you will feel intimidated or self-conscious or will you lack confidence? Why?**<br>*Probe to hear their individual experience*<br><br>**17. Is there anything else that you would like to share or are there any questions you have for me?**<br><br>**~THANK YOU FOR YOUR TIME~**
